# Simulated Drift of Dicamba and Glyphosate on Coffee Crop

**DOI:** 10.3390/plants12203525

**Published:** 2023-10-10

**Authors:** Renan Zampiroli, João Paulo Arantes Rodrigues da Cunha, Cleyton Batista de Alvarenga

**Affiliations:** Institute of Agrarian Sciences, Federal University of Uberlandia, Uberlandia 38408-100, Brazil; renanzampiroli@ufu.br (R.Z.); cleytonalvarenga@ufu.br (C.B.d.A.)

**Keywords:** *Coffea arabica* L., reduced herbicide doses, phytotoxicity, auxin herbicides

## Abstract

Weed management in areas adjacent to coffee plantations makes herbicide drift a constant concern, especially with the use of nonselective products such as dicamba. The objective of this study was to evaluate the phytotoxic effects of the herbicide dicamba alone and mixed with glyphosate as a result of simulated drift in a coffee-producing area. The study was conducted in duplicate at two different coffee cherry development stages. The study was performed with a randomized block design and a 2 × 5 + 1 factorial scheme with four replications using two herbicide spray solutions (dicamba and dicamba + glyphosate) and five low doses (0.25; 1; 5; 10; and 20%). Additionally, a control treatment without herbicide application was also employed. In this study, we evaluated the phytotoxic damage and biometric and productive parameters. Visual damages were observed with the use of dicamba and dicamba + glyphosate doses reduced by 0.25% to 5% in the first days after application. The main symptoms were new leaf epinasty, changes in the internodal distance, and plagiotropic branch curvature. Low doses led to reduced plant height and branch length. The treatments did not reduce productivity and performance but altered the physical classifications of grains.

## 1. Introduction

Coffee (*Coffea arabica* L.) is a perennial crop that can be influenced by external factors such as herbicide drift from adjacent crops. To manage weeds primarily in areas growing cereal located adjacent to coffee plantations, specific active ingredients are commonly used in Brazil. The advent of transgenic crops such as maize (*Zea mays* L.), soybean (*Glycine max* L.), and cotton (*Gossypium hirsutum* L.) increased the use of broad-spectrum herbicides [1]. However, the indiscriminate use of these products, combined with the scarce knowledge about application technology, can directly interfere with the production process and the quality of harvested grains [2].

These herbicides are often used to assist with the cultivation of soybeans. Transgenic soybean crops (tolerant to herbicides such as glyphosate, 2,4-dichlorophenoxyacetic acid (2,4-D), and glufosinate) are the most widely used herbicides in Brazil, covering approximately 68.3% of the cultivated area in 2018 [3]. A recent addition to the market, dicamba- and glyphosate-tolerant soybean cultivars, has rapidly gained significant prominence in the realm of commercial crops.

One of the challenges in dicamba application is drifting to adjacent crop areas [4], such as those growing coffee. Coffee is often produced in Brazil close to crops such as soybean, among others, which use different types of herbicides throughout their cycle, including dicamba. The vapor pressure of dicamba varies based on its formulation, with the molecule’s inherent characteristics contributing to a higher vapor pressure. This characteristic renders dicamba more susceptible to volatilization, which is influenced by factors such as meteorological conditions, application parameters (including nozzle types, adjuvants, and spray volume), and the specific commercial formulation in use [1,5,6].

Meteorological and operational factors must be considered during and after pesticide application, resulting in higher or lower drift levels, as demonstrated in several studies [7,8,9]. Drift is a phenomenon of the movement of the active ingredient away from the intended target [10]. Ref. [11] emphasized that droplets with sizes exceeding 500 µm are less prone to drift in comparison to drops smaller than 50 µm. The latter are more susceptible to evaporation and lateral displacements by wind.

Ref. [12] observed the effects of reduced dicamba and 2,4-D doses in cucumber (*Cucumis sativus* L.) and melon (*Cucumis melo* L.), reporting residual product after harvesting the fruits and severe phytotoxicity-mediated changes in both cultures. Other researchers have also reported the deleterious effects of herbicides on coffee crops, such as glyphosate, 2,4-D, and others [13,14]. However, the phytotoxic effects of dicamba on coffee plants are still unelucidated.

The effects of herbicides on nontarget plants can range from leaf chlorosis to structural deformation and even plant death, depending on the active mode of action, dose, vegetative stage of the crop, and edaphoclimatic conditions [14]. Coffee farmers often have a limited awareness of the effects of dicamba drift, often applied simultaneously with glyphosate. It is crucial for them to distinguish these effects from other alterations. Therefore, understanding the effects of dicamba and dicamba + glyphosate drift is essential for field analysis. This understanding aids in making informed decisions to mitigate issues and proactively prevent future occurrences.

Within this context, the objective of this study was to determine and evaluate the phytotoxic effects of dicamba alone and in mixture with glyphosate, under different low doses involved in simulating drift in coffee cultivation.

## 2. Results

### 2.1. Phytotoxicity in Coffee Plants

**Figure 1:**
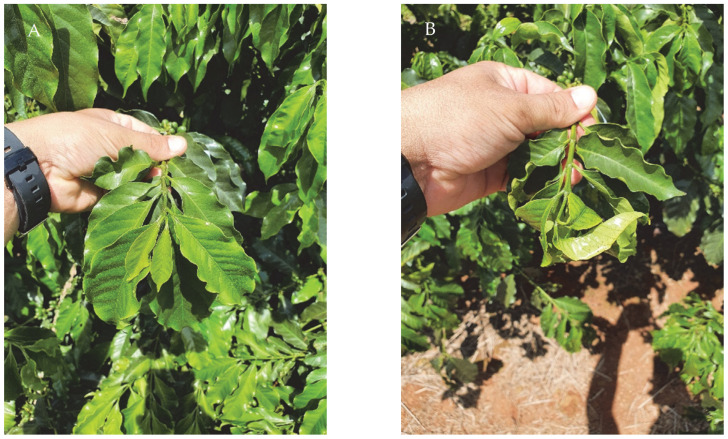

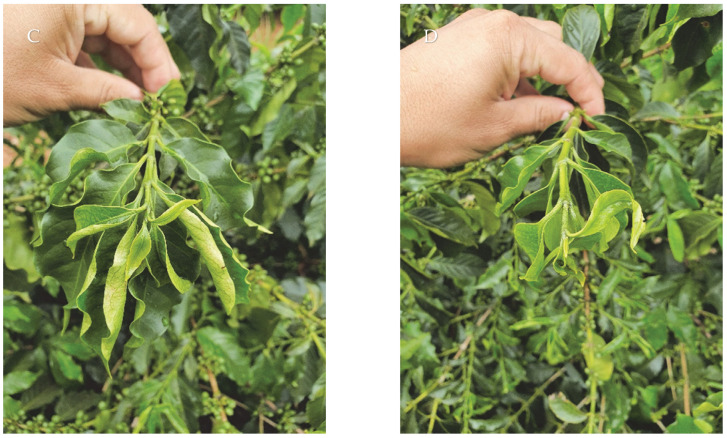
Changes caused by simulation with dicamba and dicamba + glyphosate drifts in the productive stage of the analyzed coffee crops. (**A**) Branch and leaves without changes. (**B**) Epinasty on the first leaf pair. (**C**) Epinasty and morphological changes in young leaves. (**D**) Internode elongation with drastic length and width deformation of young leaves and deformation of the plagiotropic branch curvature close to the point of growth.

The main visual changes attributed to simulation by dicamba and dicamba + glyphosate drifts in the productive stage of the analyzed coffee crops are shown in Figure 1. No leaf or branch necrosis or plant death was observed with any of the treatments.

Table 1 and Table 2 present the phytotoxicity data of Experiments I and II, respectively, showing an effect between the control and the treatments with doses reduced by more than 5%, regardless of the evaluation date. The phytotoxic effect was potentiated 7 days after treatment (DAT) when glyphosate was added to the spray. At 60 DAT, the lowest dose used was equal to that of the control in both experiments, without phytotoxic effects regardless of the spray composition. The dicamba treatment (1%) showed equivalent effects to those of the control at 90 DAT, which was not observed with added glyphosate at the same percentage. At 30 and 60 DAT, a more detrimental impact was evident with the glyphosate sprays, with similar behavior observed at 90 and 120 DAT.

**Figure 2:**
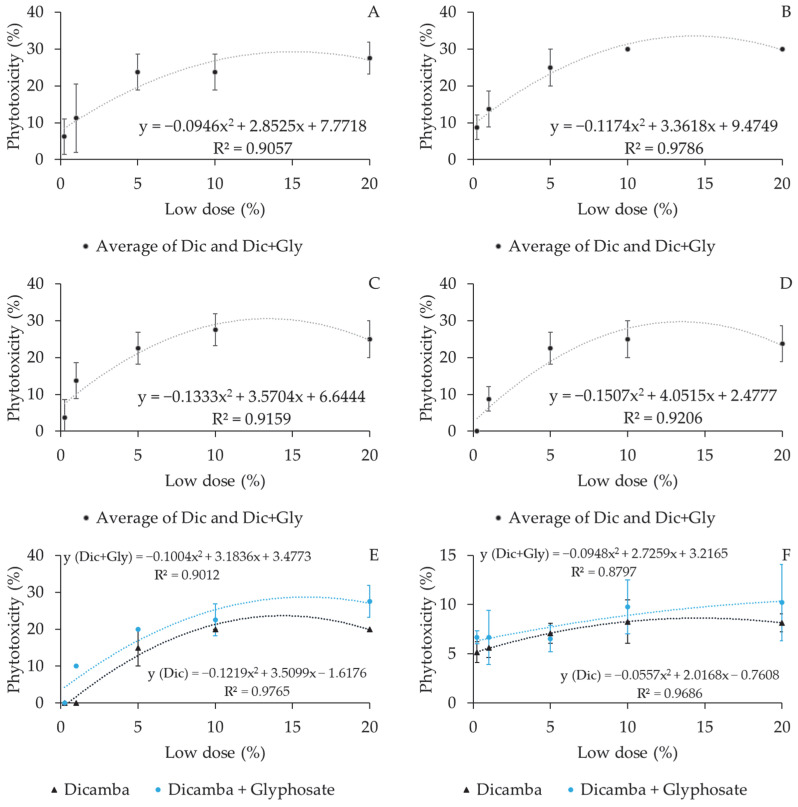
Phytotoxic behavior of reduced dicamba and dicamba + glyphosate doses at different evaluation intervals after drift simulation in coffee plants. (**A**) 7 DAT; (**B**) 15 DAT; (**C**) 30 DAT; (**D**) 60 DAT; (**E**) 90 DAT; (**F**) 120 DAT. Dic = dicamba; Dic + Gly = dicamba + glyphosate. Experiment I.

**Figure 3:**
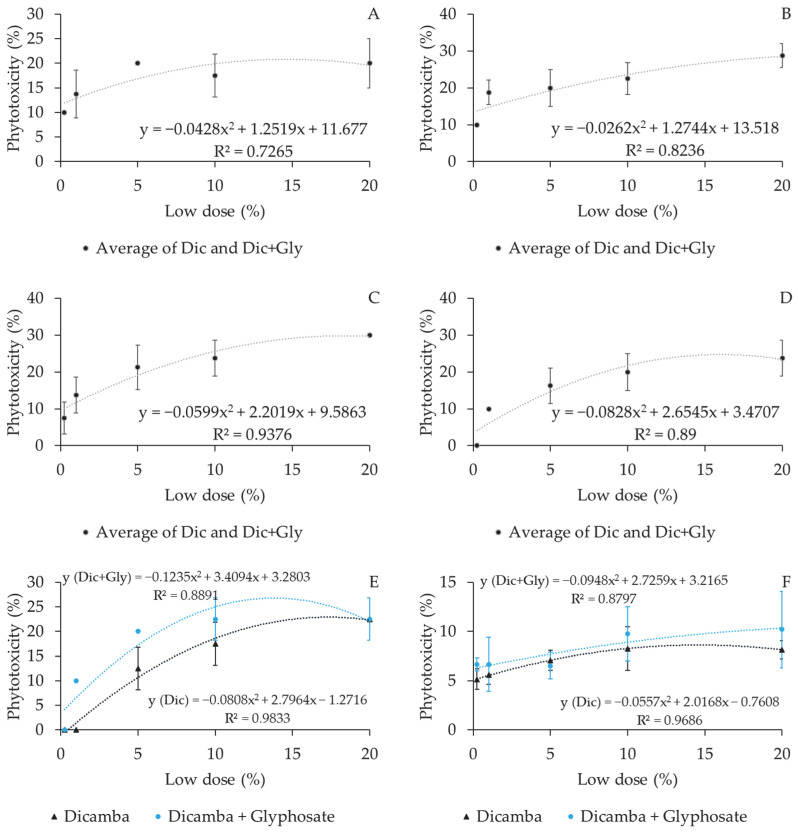
Phytotoxic behavior of reduced dicamba and dicamba + glyphosate doses at different intervals after drift simulation in coffee crops. (**A**) 7 DAT; (**B**) 15 DAT; (**C**) 30 DAT; (**D**) 60 DAT; (**E**) 90 DAT; (**F**) 120 DAT. Dic = dicamba; Dic + Gly = dicamba + glyphosate. Experiment II.

Increased doses significantly increased phytotoxicity in coffee plants, with a polynomial trend recorded during all evaluation periods. A significant difference was observed in the effects of the spray solutions tested in both experiments at 90 and 120 DAT (Figure 2 and Figure 3).

### 2.2. Biometric Variables

**Figure 4:**
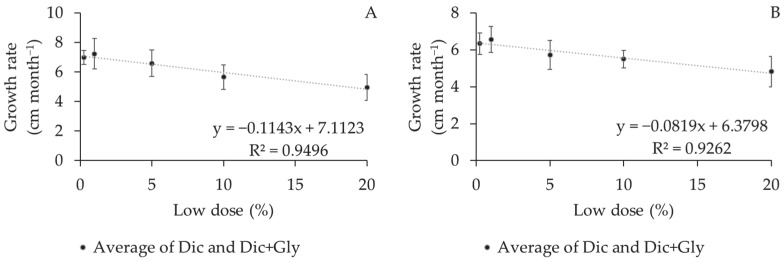
Mean height growth rate (cm month^−1^) of coffee plants subjected to low doses of herbicide sprays over a four-month period after application. (**A**) Experiment I; (**B**) Experiment II.

As for the biometric variables, no significant differences were observed in crown-diameter growth rate and the number of internodes in any of the experiments. Differences were only recorded in relation to plant height and branch length for spray solutions and the low doses used. (Table 3 and Figure 4).

Higher doses decreased plant height growth by 0.114 and 0.081 cm for each 1% dose reduction in Experiments I and II, respectively, over the four-month period (Figure 4).

In Table 4, it is observed for Experiment I the combined use of dicamba and glyphosate at doses of 10% and 20% interfered with the branch length growth rate, with an approximately 81.5% reduction observed compared to that in the control. And Experiment II showed that dicamba + glyphosate reduced the growth rate by 19.4% compared to treatments with dicamba alone, regardless of dose reduction.

**Figure 5:**
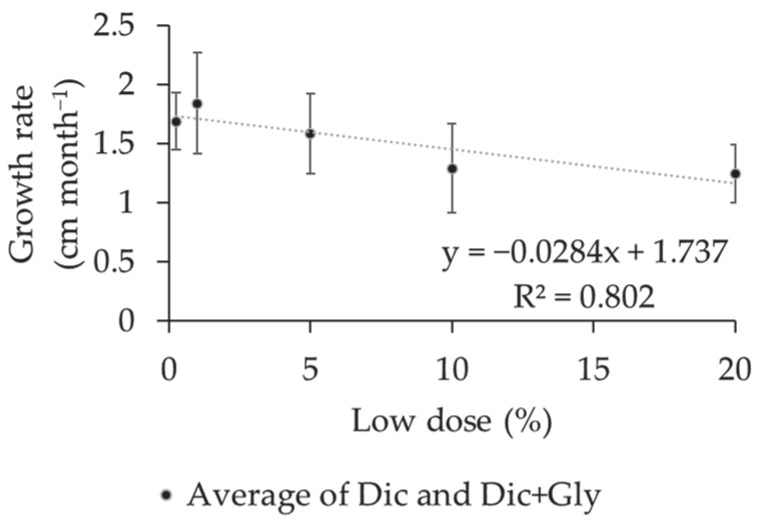
Mean growth rate of the branch length (cm month^−1^) of coffee plants subjected to different herbicide doses under conditions involving simulated drift. Experiment II.

Progressive 1% dose increases resulted in a reduction of 0.028 cm in the branch length of coffee plants in the productive stage over a four-month evaluation period, with a linear decreasing trend (Figure 5). No significant difference was observed for this variable in Experiment I.

### 2.3. Productive Parameters

As for the variable bean size and shape in Experiment I, an effect was observed between spray solutions within the Flat and Small “moca” categories. The joint application of dicamba and glyphosate in relation to dicamba alone resulted in a higher percentage of flat and small “moca” beans, with an increase of 27.5% and 16.8%, respectively. Data obtained in Experiment II show that the herbicides interfered in the classifications of Large and Small flat, with the presence of glyphosate in the spray reducing by 16.6% and increasing by 19.0% the respective classifications (Table 5).

**Figure 6:**
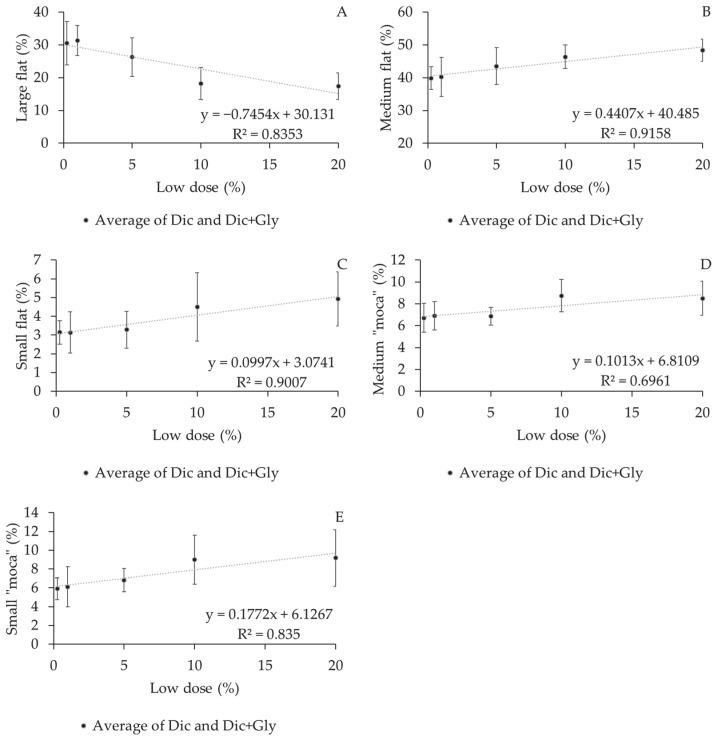
Effects of the physical classification of coffee beans subjected to different doses of dicamba and dicamba + glyphosate in a drift simulation setting: (**A**) Large flat, (**B**) Medium flat, (**C**) Small flat, (**D**) Medium “moca”, (**E**) Small “moca”. Experiment II.

The effects of increased doses in Experiment II show that only the Large flat bean classification progressively declined with increased doses, with every 1% reduction resulting in a 0.74% reduction in this classification. Thus, the increase in other classifications ranged from 0.09% for the Small flat classification to 0.44% for the Medium flat with each 1% dose increment (Figure 6). No significant difference was observed for this variable in Experiment I with the increase doses.

No differences were observed regarding the relevant parameters of productivity and performance in any of the experiments (Table 6 and Table 7).

## 3. Discussion

### 3.1. Phytotoxicity in Coffee Plants

The plants showed characteristic changes caused by auxin, as elucidated by the abnormal increase in auxin levels in the plant tissue, leading to accelerated and disordered cell division [15]. These changes were also found by [16], who evaluated the effects of 2,4-D drift on coffee and reported leaf epinasty with deformed leaf blade length and width in the first days after application. Those findings reported curvature of the orthotropic branch in plants at an early stage of development, which was not observed in this study. This change was only observed in plagiotropic branches of adult plants at doses higher than 5%, which can be possibly explained by the ability of adult plants to metabolize herbicides.

The lowest doses of dicamba (0.25% and 1%) showed a low phytotoxic effect, regardless of the mixture with glyphosate. This result can be explained by the possible metabolization by plants into less toxic or nontoxic compounds [17]. The addition of glyphosate to the spray at doses greater than 1% led to higher phytotoxic damage, which can be evidenced by the poor leaf blade formation with a trend of elongation observed 7 DAT. Glyphosate inhibits the biosynthesis of aromatic amino acids, leading to the reduced synthesis of proteins and secondary products [17].

The phytotoxicity observed in the coffee crop was already harmful in the first days after application, even at low concentrations. Batts et al. [18] evaluated simulated dicamba and dicamba + glyphosate drift, reporting deleterious effects in potato crops (*Solanum tuberosum* L.) with glyphosate in a mixture, corroborating the findings of the present study. Wells et al. [19] observed significant damage to pecan crops (*Carya illinoinensis*) at dicamba doses lower than 1% (47.9 g a.e. ha^−1^), which, as emphasized by the authors, cause significant losses to both productivity and biometric parameter values.

The results of the present study corroborate those reported by [20], who evaluated the effects of different reduced glyphosate doses on coffee cultivars. A polynomial effect was observed on phytotoxicity with increased doses. A 10% dose reduction (56 and 100 g a.e. ha^−1^ of dicamba and glyphosate, respectively) showed a trend for phytotoxic effect stabilization. Ref. [13] observed severe damage to the coffee leaf blade with simulated glyphosate drift, reporting chlorosis and leaf blade narrowing. Furthermore, these studies underscored that glyphosate translocation occurs at a slower pace in plant tissues when exposed to lower doses. This observation might elucidate the effects witnessed in the current study, where a notable disparity was evident between the spray solutions at 90 and 120 DAT. The spray comprising solely dicamba displayed minimal damage, affirming the notion that the inclusion of glyphosate in drift sprays can extend its phytotoxic effects in coffee crops. This scenario necessitates careful consideration during the assessment of phytotoxicity. Furthermore, the tolerance of different coffee cultivars to herbicides may be different with distinct symptom patterns [20].

### 3.2. Biometric Variables

Growth rate differences in Experiment I can be explained by the combined effect of dicamba and glyphosate in different action sites. Simulation with glyphosate + 2,4-D drift in *Coffea canephora* resulted in reduced growth compared to that observed with applications of isolated herbicides [14]. Ref. [15] highlighted that the exposure of sensitive plants to auxin reduced the growth rate. This observation elucidates the significant differences observed between the control group and the highest doses employed in Experiment II, particularly when supplemented with glyphosate. This combination also triggered an increase in the photosynthetic processes and nucleic acid metabolism, resulting in manifestations like leaf epinasty, branch curvature, and, in certain instances, apical meristem demise at elevated doses.

Increasing herbicide doses for crops susceptible to certain chemical groups results in deleterious effects, as reported by [20]. Furthermore, simulation with glyphosate drift at the highest doses in *Coffea arabica* cultivars resulted in reduced plant height. This effect can be potentiated in auxin-mimicking mixtures, such as dicamba. Ref. [19] evaluated the effects of the simulation of dicamba + 2,4-D drift in pecan crops (*Carya illinoinensis*) and reported severe changes in the values of the biometric parameters. Solomon et al. [21] showed that inadequate plant development and growth lead to a reduced number of branches and decreased height, diameter, and leaf area, possibly affecting photoassimilate production.

Reduced auxin doses induce hormonal disorders that impact growth points, including apical tips, plagiotropic branch ends, and developing fruits [22]. In coffee-cultivation regions, herbicide drift originating from neighboring areas can directly disrupt the biometric attributes of the crop, causing considerable harm to both present and future harvests. This interference leads to a reduction in branch growth, a parameter intrinsically tied to the productive capacity of the plants [23].

Other studies have reported the deleterious effects of auxin on biometric parameters in diverse crops [19,22,24]. However, these effects can be potentiated when mixed with herbicides in a soluble (SL) concentrate formulation, such as the glyphosate used in this study. It contains surfactants and active stabilizers, which may have contributed to the more potent deleterious effects on the coffee plant due to possible pH changes. These findings were reported by [25], with an increased auxin absorption in membranes attributed to spray acidification.

Gradual damage in a soybean crop with increasing reduced auxin doses was already observed [22,24,26], an effect that could be potentiated with the addition of glyphosate, as performed in the present study.

### 3.3. Productive Parameters

Fine-grain classifications affect coffee commercialization, with drifting increasing this percentage and reducing the value of this commodity. As mentioned by [22], auxin mediates physiological changes in susceptible crops, encompassing the effect of synthetic auxins on fruit formation. This correlation may elucidate the findings obtained in our study, given that the application took place during the initial phase of fruit development. Ref. [19] highlighted that sprays with glyphosate herbicide derived from SL formulations lead to higher synthetic auxin absorption by plant tissues. Hence, this increased absorption may be related to the decreased amounts of large flat beans and increased amounts of small flat beans. A higher quantity of large flat beans in processed coffee corresponds to a higher commercialization value, reinforcing the problem of using glyphosate in the spray. With the data obtained in both experiments (I and II), it is worth highlighting the importance of monitoring the coffee crop after verifying the phytotoxic effects attributed to dicamba alone or when mixed with glyphosate.

This reduction in the large flats may be associated with the interference of synthetic auxin on the metabolism of plants during the grain-filling process [22], directly interfering with sieve classifications. As mentioned before, fine-sieve classifications are detrimental to coffee commercialization.

Ref. [27] evaluated the effects of 2,4-D drift in coffee plants at 30-day application intervals and at different stages of fruit growth. The findings did not demonstrate any significant productivity differences, thereby corroborating the results of the present study.

Since no fruit dropping was observed during the evaluations, it is possible that the drift was not sufficient to change the load of coffee beans in post-flowering stages, as observed in the present study (load after flowering). Furthermore, [28] reported the need for additional parameters aimed at understanding the residual characteristics of the herbicides. Ref. [12] evaluated the presence of dicamba in melon and cucumber fruits and found residuals at different periods after application. In the context of coffee cultivation, this observation holds significant relevance due to the crop’s elevated economic value. With the consumer market consistently seeking heightened quality, the findings underscore the necessity for comprehensive research in this domain.

## 4. Materials and Methods

### 4.1. Characterization of the Area

The tests were conducted in Monte Carmelo, MG, Brazil, in a commercial area located at −18,884471 and −47,351121, with an altitude of 956 m above sea level. The soil was characterized as a dystrophic red oxisol [29]. According to the Köppen classification, the climate in the region is type AW—tropical hot humid, with a cold (15–16 °C) and dry winter. According to the Brazilian Institute of Meteorology, the annual precipitation and temperature averages are 1400 mm and 23 °C, respectively [30].

Drift simulation was conducted in adult, four-year-old, productive Mundo Novo coffee plants in a drip-irrigated area. The location had a population density of 3508 specimens ha^−1^, with a spacing of 3.8 m between rows and 0.75 m between individual plants, and a mean crown height and diameter of 1.69 and 1.47 m, respectively.

### 4.2. Experimental Unit and Equipment

The experiment was conducted twice (Experiment I and Experiment II), in a randomized block design, in a 2 × 5 + 1 factorial scheme, with 4 replications, considering two compositions of herbicide mixture (dicamba and dicamba + glyphosate) and five low doses (0.25; 1; 5; 10; and 20% of the recommended dose) of the respective actives and a control without application. The plots consisted of five plants, spaced by 20 plants in a row (15 m) and 7.6 m between blocks, the three central plants considered useful and the two extremities considered borders.

The sprays were prepared with a dose of 560 g acid-equivalent (a.e.) ha^−1^ of dicamba (3,6-dichloro-o-anisic acid) (Atectra^®^, BASF SA, Santo Antônio de Posse, SP, Brazil) and 1000 g a.e. ha^−1^ of glyphosate (potassium salt of N-(phosphonomethyl) glycine) (Zapp QI620, Syngenta SA, São Paulo, SP, Brazil) (Table 8).

The application procedure was carried out following the methodology adapted from [22]. A total of 2 L volume of the sprays was prepared, which was necessary to fill the polyethylene terephthalate bottles used as reservoirs of the CO_2_ pressurized backpack sprayer (Herbicat, São Paulo, SP, Brazil). The application bar was fitted with a nozzle featuring a GRD 12002 fan-type tip (Hypro Pentair, MN, USA). This nozzle had a jet opening angle of 120° and was operated at a designated flow rate of 0.757 L min^−1^ under a pressure of 280 kPa. This configuration was chosen to generate fine to very fine droplets, as specified by the manufacturer.

**Figure 7:**
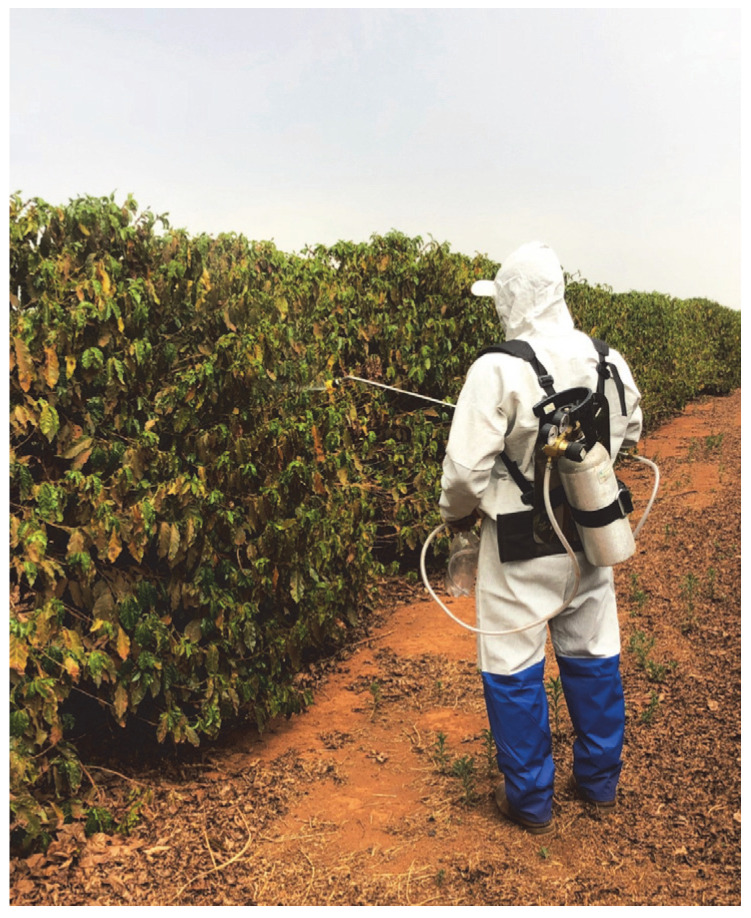
Pressurized knapsack sprayer used for the treatment applications.

The tip was maintained at a distance of 0.5 m away from the coffee crown during application to cover the entire leaf area with an up and down movement on both sides of the plants. The working condition was maintained for an application rate of 150 L ha^−1^, with approximately 42.75 mL plant^−1^ (application rate divided by number of plants per ha) (Figure 7).

The meteorological conditions were monitored in real time during both experiments using a thermo-hygro-anemometer (LM-800, Instrutherm, São Paulo, SP, Brazil) (Table 9).

**Figure 8:**
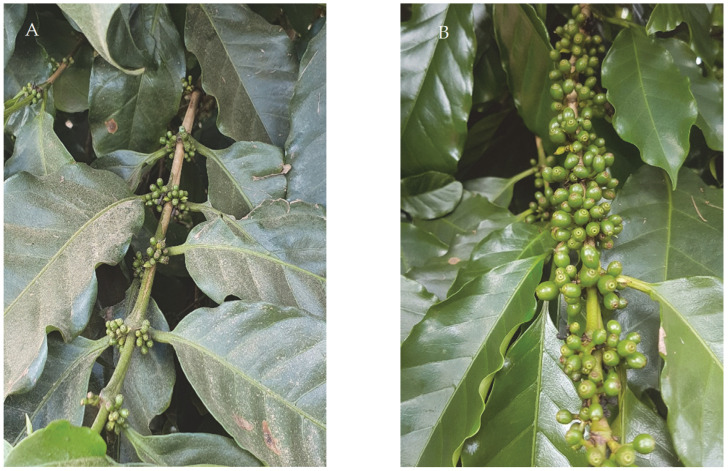
Fruit development stage at the time of treatments. (**A**) Formation stage. (**B**) Expansion stage.

The sprays were applied in November (Experiment I) and December (Experiment II) 2020, corresponding to two stages of coffee fruit development, namely, the formation stage, when the cherries show no visible growth, and the expansion stage, when the cherries enlarge with a characteristic size and shape [31] (Figure 8).

### 4.3. Evaluated Parameters

Phytotoxicity was assessed after application using a grade scale adapted from [20], with scores from 0% to 100%. Here, the scores of “0”, “10–30%”, “40–60%”, “70–100%”, and “100%” corresponded to no changes, alterations in the visual shape of the leaf blade, damage involving tissue necrosis, apical bud death and extensive branch necrosis, and complete plant mortality, respectively. The plants were evaluated at 7, 15, 30, 60, 90, and 120 DAT.

In addition to the phytotoxicity analysis, the growth rate in terms of biometric variables was determined based on plant height, canopy diameter, branch length, and the number of internodes over a four-month evaluation period. The following parameters were assessed at the initiation and conclusion of the experiment and were calculated as the difference in growth over the designated time period. Here, the growth rate was computed using the following equation:

(1) $$\mathrm{Grow}\;\mathrm{rate}=\frac{\mathrm{Final}\;\mathrm{growth}-\mathrm{Initial}\;\mathrm{growth}}{\mathrm{Time}\;(\mathrm{four}\;\mathrm{months})}$$

Plant height was measured utilizing a rigid measuring tape, capturing the height of the three central plants within the plot from the ground level to the apical bud. The crown diameter was determined as the mean diameter of the canopy, which was measured between the ends of plagiotropic branches in the middle third of the plant. The length of the branch was measured using a flexible measuring tape in the middle third of the three central plants on both sides of the plant from the point of insertion in the orthotropic branch to the point of growth of the branch. The number of internodes was directly counted in the same branches where branch length was determined. At the end of the experiment, productive parameters (physical classification of coffee beans, productivity (bag ha^−1^), and performance (L kg^−1^)) were evaluated following the methodology adapted from [32,33]. For better control, the plots were harvested manually when the percentage of green cherries was lower than 20%, following the methodology adapted from [34]. After harvesting the total volume produced in each plot, a 6 L sample was separated for post-harvest analyses.

The 6 L sample was placed in polyethylene net bags and dried on a suspended terrace until the atmosphere showed 12% humidity, which was measured using a moisture meter (G800, Gehaka, São Paulo, SP, Brazil). Then, the dry coffee mass was determined using a semi-analytical scale (BL3200H, Shimadzu, Barueri, SP, Brazil), and the volume of the sample was measured with a 1000 mL graduated cylinder. Subsequently, the samples were processed using a processor (DRC-1, Carmomaq, Espírito Santo do Pinhal, SP, Brazil), and mass and volume were determined again. The moisture of the samples was then determined to correct the masses.

Based on the ratio of the volume of the sample (6 L) and the mass of the processed sample, the production per plot was determined and extrapolated to determine productivity in bags of 60 kg ha^−1^. Performance parameters (liters kg^−1^) were also evaluated as follows: (a) field coffee: volume of ripe coffee harvested to produce 1 kg of processed coffee, and (b) dry coffee: volume of dry coffee used to produce 1 kg of processed coffee.

The physical classification of the size and shape of the beans was performed following the methodology described in the Brazilian Legislation [35] using a sample of 100 g of processed coffee and a set of circular (18, 17, 16, 15, 14, and 13/64ths of an inch) and oblong sieves (13, 12, 11, 10, 9, and 8/64ths of an inch). Circular sieves retained flat beans, and oblong sieves separated the “moca” beans. Hence, the separation was performed, and the following categories were evaluated: (a) Large flat—sieves 18 and 17; (b) Medium flat—sieves 16 and 15; (c) Small flat—sieves 14 and smaller; (d) Large “moca”—sieves 13, 12, and 11; (e) Medium “moca”—sieves 10; and (f) Small “moca”: sieve 9 and smaller.

### 4.4. Statistical Analysis

After data collection, residual normality and variance homogeneity were determined using the Shapiro–Wilk and Bartlett tests, respectively. Subsequently, the data were subjected to an analysis of variance (ANOVA). Regression analysis was performed to examine the low doses. An F-test was used to compare the means of the qualitative factors, and a Dunnett’s test was performed to compare the means of the qualitative factors w the control. R statistical analysis software (v.3.6.0) [36] was used for these analyses.

## 5. Conclusions

Dicamba and dicamba + glyphosate drift induced visual damages to coffee plants in the first days after application. The intensity of this effect varied with the dose and the crop stage.

The main changes observed are outlined as follows: new leaf epinasty and internode distance and plagiotropic branch curvature changes.

The increase in herbicide doses in the simulated drift led to reduced plant height and branch length.

The effects of simulation with dicamba and dicamba + glyphosate drift on coffee plants did not reduce productivity and performance during the productive stage in the present study. However, changes were observed in the physical classifications of beans (flat and “moca”) according to the stages of coffee development.

## Figures and Tables

**Table 1:** Phytotoxicity (%) in coffee plants after herbicide treatments and at different low doses. Experiment I.

Low Dose (%)	DAT (Days)											
	7		15		30		60		90		120	
	C1	C2	C1	C2	C1	C2	C1	C2	C1	C2	C1	C2
0.25	5.0 *	7.5 *	7.5 *	10.0 *	2.5	5.0 *	0.0	0.0	0.0 A	0.0 A	0.0 A	0.0 A
1	5.0 *	17.5 *	10.0 *	17.5 *	10.0 *	17.5 *	7.5 *	10.0 *	0.0 A	10.0 B*	0.0 A	10.0 B*
5	20.0 *	27.5 *	22.5 *	27.5 *	20.0 *	25.0 *	20.0 *	25.0 *	15.0 A*	20.0 B*	10.0 A*	15.0 B*
10	20.0 *	27.5 *	30.0 *	30.0 *	25.0 *	27.5 *	25.0 *	25.0 *	20.0 A*	22.5 A*	12.5 A*	20.0 B*
20	25.0 *	30.0 *	30.0 *	30.0 *	20.0 *	30.0 *	20.0 *	27.5 *	20.0 A*	27.5 B*	17.5 A*	20.0 A*
Mean	15.0 A	16.5 B	20.0 A	23.0 B	20.1 A	21.0 B	14.5 A	17.5 B	11.0	16.0	8.0	13.0
Control	0		0		0		0		0		0	
CV (%)	23.9		16.7		21.2		19.7		14.3		16.3	

Means followed by capital letters in the line differ between spray solutions (C1 and C2) using an F-test (*p* < 0.05). Means followed by * correspond to differences with respect to the control, as assessed by a Dunnett’s test (*p* < 0.05). C1 = dicamba; C2 = dicamba + glyphosate. CV = coefficient of variation.

**Table 2:** Phytotoxicity (%) in coffee plants after herbicide treatments and at different low doses. Experiment II.

Low Dose (%)	DAT (Days)											
	7		15		30		60		90		120	
	C1	C2	C1	C2	C1	C2	C1	C2	C1	C2	C1	C2
0.25	10.0 *	10.0 *	10.0 *	10.0 *	7.5 *	7.5 *	0.0	0.0	0.0 A	0.0 A	0.0 A	0.0 A
1	12.5 *	15.0 *	17.5 *	20.0 *	10.0 *	17.5 *	10.0 *	10.0 *	0.0 A	10.0 B	0.0 A	10.0 B*
5	20.0 *	20.0 *	27.5 *	27.5 *	17.5 *	25.0 *	12.5 *	20.0 *	12.5 A*	20.0 B*	10.0 A*	15.0 B*
10	17.5 *	17.5 *	20.0 *	25.0 *	20.0 *	27.5 *	17.5 *	22.5 *	17.5 A*	22.5 A*	12.5 A*	20.0 B*
20	20.0 *	20.0 *	30.0 *	27.5 *	30.0 *	30.0 *	22.5 *	25.0 *	22.5 A*	22.5 A*	17.5 A*	20.0 A*
Mean	16.0	16.5	21.0	22.0	17.0 A	21.5 B	12.5 A	15.5 B	10.5	15.0	8.0	13.0
Control	0		0		0		0		0		0	
CV (%)	24.5		19.6		22.1		28.0		30.0		27.4	

Means followed by capital letters in the line differ between spray solutions (C1 and C2) using an F-test (*p* < 0.05). Means followed by * correspond to differences with respect to the control, as assessed by a Dunnett’s test (*p* < 0.05). C1 = dicamba; C2 = dicamba + glyphosate. CV = coefficient of variation.

**Table 3:** Growth rate of the heights (cm month^−1^) of coffee plants subjected to simulation with dicamba and dicamba + glyphosate drifts at different low doses.

Low Dose (%)	Experiment I		Experiment II	
	C1	C2	C1	C2
0.25	6.8	7.1	6.5	6.2
1	7.2	7.2	6.9	6.2
5	7.3	5.9	6.0	5.4 *
10	6.0	5.3	5.7	5.3 *
20	5.8	4.1	5.6 *	4.0 *
Mean	6.6 A	5.9 B	6.1	5.5
Control	6.6		7.2	
CV (%)	12.4		13.3	

Means followed by capital letters in the line differ between spray solutions (C1 and C2) using an F-test (*p* < 0.05) within the respective experiments. Means followed by * correspond to differences with respect to the control, as assessed by a Dunnett’s test (*p* < 0.05) within the respective experiments. C1 = dicamba; C2 = dicamba + glyphosate. CV = coefficient of variation.

**Table 4:** Growth rate of the branch length (cm month^−1^) of coffee plants subjected to simulated dicamba and dicamba + glyphosate drift at different low doses.

Low Dose (%)	Experiment I		Experiment II	
	C1	C2	C1	C2
0.25	1.9	2.0	1.5	1.8
1	1.8	2.5	2.1	1.6
5	2.2	1.7	1.8	1.4
10	1.7	1.5 *	1.4	1.1
20	1.8	1.4 *	1.4	1.1
Mean	1.9	1.8	1.7 A	1.4 B
Control	2.7		1.5	
CV (%)	26.3		19.9	

Means followed by capital letters in the line differ between spray solutions (C1 and C2) using an F-test (*p* < 0.05) within the respective experiments. Means followed by * correspond to differences with respect to the control, as assessed by a Dunnett’s test (*p* < 0.05) within the respective experiments. C1 = dicamba; C2 = dicamba + glyphosate. CV = coefficient of variation.

**Table 5:** Physical classification of coffee beans (%) subjected to simulated dicamba and dicamba + glyphosate drift.

Spray Solutions	Experiment I		Experiment II	
	Small Flat	Small “Moca”	Large Flat	Small Flat
C1	3.74 a	6.2 a	26.7 a	3.4 a
C2	5.2 b	7.4 b	22.9 b	4.2 b
Control	2.4	6.5	28.7	3.5
CV (%)	46.4	27.2	21.4	31.7

Means followed by different lowercase letters in the column for each classification differ from each other using an F-test (*p* < 0.05) within the respective experiments. C1 = dicamba; C2 = dicamba + glyphosate. CV = coefficient of variation.

**Table 6:** Mean productivity (bag ha^−1^) of coffee plants subjected to simulated dicamba and dicamba + glyphosate drift at different low doses.

Low Dose (%)	Experiment I		Experiment II	
	C1	C2	C1	C2
0.25	77.6	71.3	68.9	78.5
1	85.0	79.0	69.9	73.0
5	74.9	67.2	78.4	72.9
10	72.1	71.1	74.4	70.6
20	79.5	72.9	69.9	73.9
Mean	77.8	72.4	72.3	73.8
Control	77.5		73.1	
CV (%)	23.4		13.0	

C1 = dicamba; C2 = dicamba + glyphosate. CV = coefficient of variation.

**Table 7:** Performance (L kg^−1^) of coffee subjected to simulated dicamba and dicamba + glyphosate drift at different low doses.

Low Dose (%)	Field Coffee				Dry Coffee			
	Experiment I		Experiment II		Experiment I		Experiment II	
	C1	C2	C1	C2	C1	C2	C1	C2
0.25	9.1	8.5	8.0	8.6	5.6	5.5	5.3	5.3
1	7.8	8.3	8.6	8.1	5.3	5.2	5.6	5.5
5	8.4	8.3	8.5	8.8	5.6	5.9	5.8	5.5
10	8.9	8.2	8.4	8.2	5.6	5.6	5.7	5.6
20	8.2	8.8	8.4	8.3	5.5	5.7	5.5	5.7
Mean	8.5	8.4	8.4	8.4	5.5	5.6	5.6	5.5
Control	9.3		8.3		5.4		5.6	
CV (%)	8.9		9.3		5.6		3.9	

C1 = dicamba; C2 = dicamba + glyphosate. CV = coefficient of variation.

**Table 8:** Low doses of herbicides (g a.e. ha^−1^) used in the drift simulation.

Low Dose (%)	Dicamba	Glyphosate
0.25	1.4	2.5
1	5.6	10
5	28	50
10	56	100
20	112	200

a.e. = acid equivalent.

**Table 9:** Average meteorological conditions during the proposed treatments.

Experiment	Temperature (°C)	Relative Humidity (%)	Wind Speed (m s^−1^)
I	23.6	85	0.8
II	26.7	71	1.9

## Data Availability

All data included in the main text.
